# Methylone, a rapid acting entactogen with robust anxiolytic and antidepressant-like activity

**DOI:** 10.3389/fpsyt.2022.1041277

**Published:** 2023-01-10

**Authors:** Jennifer Warner-Schmidt, Christopher Pittenger, Martin Stogniew, Blake Mandell, Sarah J. Olmstead, Benjamin Kelmendi

**Affiliations:** ^1^Transcend Therapeutics, New York, NY, United States; ^2^Department of Psychiatry, Yale School of Medicine, New Haven, CT, United States; ^3^Clinical Neurosciences Division, United States Department of Veterans Affairs, National Center for PTSD, West Haven, CT, United States

**Keywords:** empathogen, serotonin, SERT, PTSD, depression, anxiety, MDMA

## Abstract

**Introduction:**

Selective serotonin reuptake inhibitor (SSRI) antidepressants represent first-line pharmacological treatment for a variety of neuropsychiatric illnesses, including major depressive disorder (MDD), anxiety, and post-traumatic stress disorder (PTSD), which show high rates of comorbidity. SSRIs have a delayed onset of action. Most patients do not show significant effects until 4–8 weeks of continuous treatment, have impairing side effects and as many as 40% of patients do not respond. Methylone (3,4-methylenedioxy-*N*-methylcathinone; MDMC, βk-MDMA, M1) is a rapid-acting entactogen that showed significant benefit in a clinical case series of PTSD patients and was well-tolerated in two Phase 1 studies of healthy volunteers. Based on these early observations in humans, in the current study we tested the hypothesis that methylone has antidepressant-like and anxiolytic effects in preclinical tests.

**Methods:**

For all studies, 6–8-week-old male Sprague Dawley rats (*N* = 6–16) were used. We employed the Forced Swim Test (FST), a classic and widely used screen for antidepressants, to explore the effects of methylone and to probe dose-response relationships, durability of effect, and potential interactions with combined SSRI treatment. We compared the effect of methylone with the prototypical SSRI fluoxetine.

**Results:**

Three doses of fluoxetine (10 mg/kg) given within 24 h before FST testing caused a 50% reduction in immobility compared with controls that lasted less than 24 h. In contrast, a single dose of methylone (5–30 mg/kg) administered 30 min prior to testing produced a rapid, robust, and durable antidepressant-like response in the FST, greater in magnitude than fluoxetine. Immobility was reduced by nearly 95% vs. controls and effects persisted for at least 72 h after a single dose (15 mg/kg). Effects on swimming and climbing behavior in the FST, which reflect serotonergic and noradrenergic activity, respectively, were consistent with studies showing that methylone is less serotoninergic than MDMA. Fluoxetine pretreatment did not change methylone’s antidepressant-like effect in the FST, suggesting the possibility that the two may be co-administered. In addition, methylone (5–30 mg/kg) exhibited anxiolytic effects measured as increased time spent in the center of an open field.

**Discussion:**

Taken together, and consistent with initial clinical findings, our study suggests that methylone may have potential for treating depression and anxiety.

## Introduction

Major depressive disorder (MDD) and anxiety are debilitating diseases with lifetime prevalences of 20.8 and 28.8%, respectively, and a comorbidity rate estimated to be as high as 60% (1). Antidepressants remain a first-line treatment for these and many other central nervous system (CNS) disorders. The development of rapid-acting psychoactive therapeutics offers advantages over traditional slow-acting antidepressants (SAADs). SAADs, such as selective serotonin reuptake inhibitors (SSRIs) and tricyclic antidepressants, have a slow onset of action, requiring as long as 4–8 weeks of continuous treatment for potential clinical benefit and have significant side-effects that may hinder compliance with treatment; even when optimally administered, as many as 40% of individuals do not respond (2, 3). Rapid-acting antidepressants (RAADs) offer a number of advantages over SAADs, the first of which is potentially improved efficacy, but also the possibility of improved compliance with a treatment due to rapid onset of action, a shorter-term treatment duration, and durable therapeutic effects that do not require daily dosing (4, 5). Esketamine is the only RAAD that has been FDA-approved as an adjunct therapy for treatment-resistant depression (6)^®^ [package insert]. Titusville, NJ, USA: (Janssen Pharmaceuticals, Inc., 2020), but other drugs in development include psilocybin and 3,4-methylenedioxymethamphetamine (MDMA), which appear to show clinical benefit for the treatment of MDD and post-traumatic stress disorder (PTSD), respectively (4, 7, 8).

Methylone (also known as 3,4-methylenedioxy-*N*-methylcathinone, MDMC, βk-MDMA, and M1) is an entactogen and a beta-ketone analog of MDMA currently in development for the treatment of PTSD. Methylone was synthesized over 25 years ago (9), but the literature describing its properties is relatively sparse, focused largely on *in vitro* studies or binge-dosing regimens that mimic its illicit use. Methylone shares some chemical and pharmacological properties with MDMA, but also has some differences. For example, methylone is a serotonin (5HT), norepinephrine (NE), and dopamine (DA) reuptake inhibitor and releaser like MDMA, but with 3–4× lower potency for inhibition of serotonin uptake (10).

To date, clinical experience with methylone has been described in four studies, which demonstrate that it is well tolerated, produces a milder range of effects compared with MDMA (11, 12), and alleviates symptoms of PTSD in a retrospective clinical case series (13). It is notable that MDD and anxiety show high rates of comorbidity with PTSD (14), and SSRIs are used to treat all three disorders, suggesting that the reported effects of methylone in PTSD (13) may translate to therapeutic benefit in multiple CNS disorders. In fact, a second retrospective clinical case series suggests methylone may have RAAD effects in patients with MDD (15).

Based on the initial clinical reports of methylone’s potentially therapeutic activity in PTSD (13) and MDD (15), as well as effects of structurally similar MDMA in clinical (16) and preclinical studies (17, 18), we hypothesized that methylone would show rapid-onset antidepressant and anxiolytic activity in preclinical behavioral tests. To test this, we evaluated the effect of methylone or fluoxetine compared to vehicle treated controls in the forced swim test (FST) and the open field test (OFT) in rats, probing its dose response, duration of activity, and interaction with combined SSRI treatment. Pharmacokinetics in plasma and brain at active doses were also assessed.

## Materials and methods

### Pharmacokinetics (PK)

Pharmacokinetics studies were carried out at WuXi Apptec, Inc. (Cranbury, NJ, USA) using standard protocols. All animal use and procedures were approved by the WuXi Apptec, Inc., IACUC. Briefly, terminal blood and brain samples were collected from 6 to 8 week old male Sprague Dawley rats (Hilltop Lab Animals, Inc., Scottsdale, PA, USA) at 0.25, 0.5, 1, 2, 4, and 8 h (*N* = 3 per group per time point) for the determination of plasma concentrations of methylone following intraperitoneal (IP) dosing of 5, 10, or 15 mg/kg methylone. Since these were terminal collections, three independent rats per group were used for each time point in a single PK curve from which PK parameters (C_max_, AUC, etc.) were generated. Samples were collected into K_2_EDTA tubes on ice and centrifuged at 3000 × *g* at 4°C for 5 min within 30 min of collection. Plasma samples were stored at −80°C until analysis by liquid chromatography tandem mass spectrometry (LC-MS/MS). The bioanalytical assay provided a lower limit of quantification (LLOQ) of 1 ng/mL and an upper limit of quantification (ULOQ) of 3000 ng/mL for methylone. The plasma concentration-time data were analyzed using Phoenix WinNonlin (version 8.3) to characterize the PK properties of the analyte. The non-compartmental analysis model and the linear/log trapezoidal method were applied to the calculation of the PK parameters.

### Behavioral testing

#### Animals

Male Sprague Dawley rats (Charles River Laboratories) weighing 180–200 g on arrival, were used for all behavioral studies, which took place at Melior Discovery (Exton, PA, USA). Rats acclimated to their home cages for at least 1 week before testing, were maintained in a controlled environment on a 12 h light/dark cycle, with no more than 2 rats per cage. Animals received *ad libitum* access to standard rodent chow and water and were assigned randomly to treatment groups. All animal use and procedures were in accordance with established protocols approved by the Melior IACUC committee, Melior Standard Operation Procedures (SOP), and Transcend Therapeutics. Distinct cohorts of animals were used for each experiment/Figure presented in this manuscript.

#### Drug treatments

Methylone HCl (0.5–30 mg/kg; Cayman Chemical) or Fluoxetine HCl (10 mg/kg; Sigma-Aldrich) were formulated in sterile 0.9% saline vehicle before IP administration. Control animals received saline vehicle. Methylone or saline was administered 30 min prior to FST or OFT testing sessions. Fluoxetine or saline were administered 23.5, 5, and 1 h prior to testing, consistent with previous studies (19).

#### Forced swim test (FST)

All studies were performed and scored by an experimenter blind to treatment group according to standard protocols at Melior Discovery (Exton, PA, USA) and based on published “modified FST” procedures (20). Briefly, rats were placed in a circular plexiglass container (29.2 cm diameter, 49.5 cm height) filled with water to a depth of 30 cm so rats could not support themselves by touching the bottom of the tank. Water was maintained at 22–25°C and was changed for every animal. Day 1 (Training) consisted of a 15 min acclimation trial, and Day 2 (Testing, 24 h later) consisted of the 5 min test. A time sampling procedure was employed where animals were observed every 5 s for the duration of the test session (60 counts or 5 min) and scored for immobility (defined as the failure to struggle), swimming (defined as a circular movement around the tank), or climbing (defined as an upward escape behavior). Data are expressed as the percent time spent immobile, swimming, or climbing for the 5-min testing session (e.g., the number of immobility counts divided by 60). Therefore, to extrapolate these values to time spent immobile in minutes, one can multiply the percent time immobile by 5 min. For example, if an animal shows 80% time spent immobile, this means that 4 min (0.8 × 5) of the 5-min testing session were spent immobile.

Separate vehicle control groups were used for methylone (one injection) and fluoxetine (three injections) since the stress of repeated drug injections can increase immobility time in the FST, accounting for the small difference between those two control groups.

#### Open field test (OFT)

All studies were performed by an experimenter blind to treatment group and according to standard protocols at Melior Discovery (Exton, PA, USA). The OFT was used to assess both locomotor activity and anxiety-like behavior. The OFT was run in a standard rat OFA chamber (17′′ × 17′′ × 12′′, Med Associates). After habituation to the testing room and drug injection, rats were assessed for 30 min in the OFT using an automated activity monitoring system (MedAssociates). Locomotor activity was measured by recording the total ambulatory distance traveled (cm), reported in 5-min bins for the duration of the 30 min testing period. The center of the open field was defined as a 12′′ square, a predefined center setting on the MedAssociates analysis software. Time spent in the center of the open field, an anxiolytic measure, was also recorded for the 30 min testing period.

#### Statistical analysis

All data are presented as the mean ± SEM. Differences between two groups were determined by unpaired *t*-test, differences between more than two groups were determined by one-way ANOVA and *post hoc* multiple comparison test noted in Figure Legends. When there were two different variables (drug × time), differences were determined by two-way ANOVA and Bonferroni’s *post hoc* multiple comparison test unless otherwise noted. A *p*-value ≤ 0.05 indicated statistical significance. All analyses were completed using Graphpad Prism software version 9.3.1 (San Diego, CA, USA).

## Results

### Effects of methylone on antidepressant-like activity

We employed the FST, a classic and commonly used preclinical behavioral paradigm to screen drugs for antidepressant-like activity, to test the effect of methylone. Antidepressants consistently reduce immobility time in the FST, and accompanying increases in climbing or swimming behaviors reflect noradrenergic or serotonergic involvement, respectively (19). Multiple classes of antidepressants, including SSRIs like fluoxetine and more recent RAADs like ketamine, psilocybin, and MDMA have all been reported to reduce immobility in the FST (17, 19, 21–23).

**FIGURE 1:**
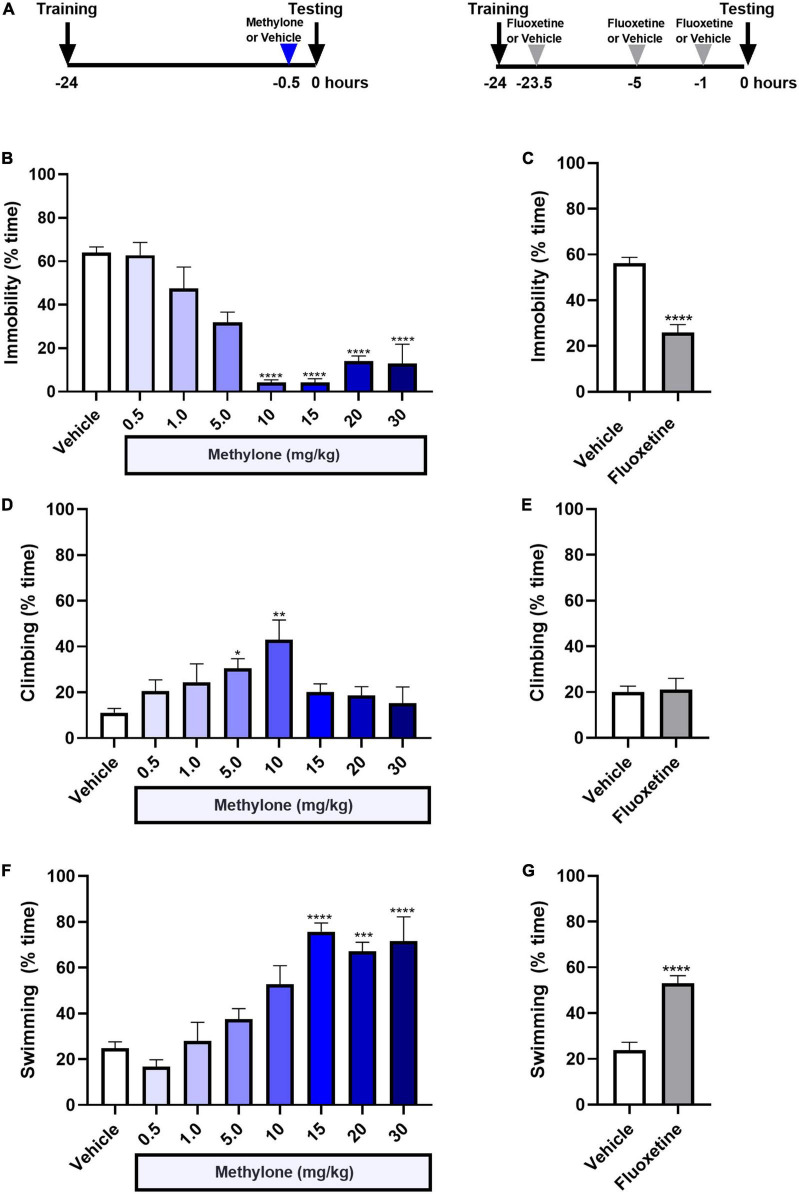
Methylone has a rapid-acting and robust antidepressant-like response in the forced swim test. **(A)** Schematic shows experimental design. Methylone (0.5–30 mg/kg, IP) or saline (Vehicle) was administered 30 min prior to testing. Fluoxetine (10 mg/kg, IP) or saline (Vehicle) were administered 23.5, 5, and 1 h prior to testing. Quantification of the percent time spent **(B,C)** immobile, **(D,E)** climbing, or **(F,G)** swimming during the 5-min test session is shown. Data are presented as means ± SEM. *p < 0.05, **p < 0.01, ***p < 0.001, ****p < 0.0001 vs. Vehicle control group, Bonferroni’s *post-hoc* test; *N* = 6–16 per group.

Here, rats received a single injection of methylone (0.5–30 mg/kg, IP) or saline (Vehicle) 30 min before testing in the FST (Figure 1A). Results are presented as the percent time spent immobile during the 5-min testing session. A low dose of methylone (5 mg/kg) significantly reduced the percent immobility time to 31.9 ± 4.7% compared to Vehicle controls 63.9 ± 2.7%. Moderate doses of methylone (10–15 mg/kg) robustly reduced immobility to 4.2 ± 1.3% compared with Vehicle controls 63.9 ± 2.7%. [Figure 1B, *F*_(7,67)_ = 23.32, *p* < 0.0001]. There was a trend toward less reduction in immobility at the highest doses of methylone (20–30 mg/kg), suggesting a possible U-shaped dose-response curve, although this did not reach statistical significance. As a positive control and comparator, the SSRI fluoxetine (10 mg/kg, IP) or saline (Vehicle) were administered 23.5, 5, and 1 h before testing, based on previous studies (24). The effect of three doses of fluoxetine was comparable in magnitude to a single low (5 mg/kg) dose of methylone in the FST, roughly a 50% change from their respective vehicle control group [Figure 1C, *t*_(12)_ = 7.149, *p* < 0.0001]. Climbing behavior was increased only by low to mid doses of methylone (5–10 mg/kg) compared with controls [Figure 1D, *F*_(7,67)_ = 3.696, *p* < 0.01] and was unaffected by fluoxetine [Figure 1E, *t*_(12)_ = 0.2133, *p* = 0.8, n.s.]. Swimming behavior was significantly increased by mid to high doses of methylone [Figure 1F, *F*_(7,67)_ = 14.44, *p* < 0.0001] and, as expected, also by fluoxetine [Figure 1G, *t*_(12)_ = 5.844, *p* < 0.0001].

**FIGURE 2:**
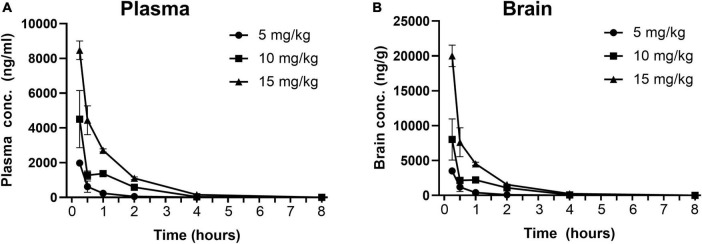
Concentration-time profiles of methylone in plasma and brain following a single dose. Methylone (5, 10, 15 mg/kg, IP) was administered and terminal blood and brain samples were collected 0.25, 5, 1, 2, 4, and 8 h post-dose. Concentrations of methylone detected in the **(A)** plasma and **(B)** brain are shown. Data are presented as means ± SEM. *N* = 3 per time point per group.

Pharmacokinetic profiles of methylone in plasma and brain were determined at efficacious doses in the FST. Rats were injected with methylone (5, 10, or 15 mg/kg) and terminal blood and brain samples were collected 0.25, 5, 1, 2, 4, and 8 h post-dose. Concentrations of methylone in plasma and brain are plotted in Figure 2. Following a single IP administration of methylone at 5, 10, or 15 mg/kg, the peak plasma concentrations (C_max_) of methylone were 1983, 4507, and 8470 ng/mL, respectively. The C_max_ was achieved 15 min (T_max_) post-dose. The area under the plasma concentration-time curve from time 0 to the last quantifiable time (AUC_0–last_) of methylone was 908, 3242, and 7320 h/mL, respectively. The terminal elimination half-life (T_1/2_) of methylone was 0.6–0.8 h and the mean residence time from time 0 to the last quantifiable time (MRT_0–last_) was 0.7–1.13 h. The brain to plasma AUC ratio was approximately 1.8, demonstrating that methylone effectively crossed the blood-brain barrier. Selected plasma and brain PK parameters are presented in Table 1.

**TABLE 1:** Pharmacokinetic properties of methylone (5, 10, 15 mg/kg, IP) in rat brain and plasma.

PK parameters of methylone						
**PK parameters**	**5 mg/kg**		**10 mg/kg**		**15 mg/kg**	
	**Mean brain**	**Mean plasma**	**Mean brain**	**Mean plasma**	**Mean brain**	**Mean plasma**
C_max_ (ng/mL or ng/g)	3513	1983	8021	4507	20013	8470
T_1/2_ (h)	0.691	0.598	0.607	0.674	0.884	0.811
T_last_ (h)	4.00	4.00	4.00	8.00	8.00	8.00
AUC_0–last_ (ng.h/mL or ng.h/g)	1661	908	5576	3242	13201	7320
MRT_0–last_ (h)	0.696	0.666	1.03	1.07	1.01	1.13
AUC ratio (brain: plasma)	1.83	–	1.72	–	1.80	–

**FIGURE 3:**
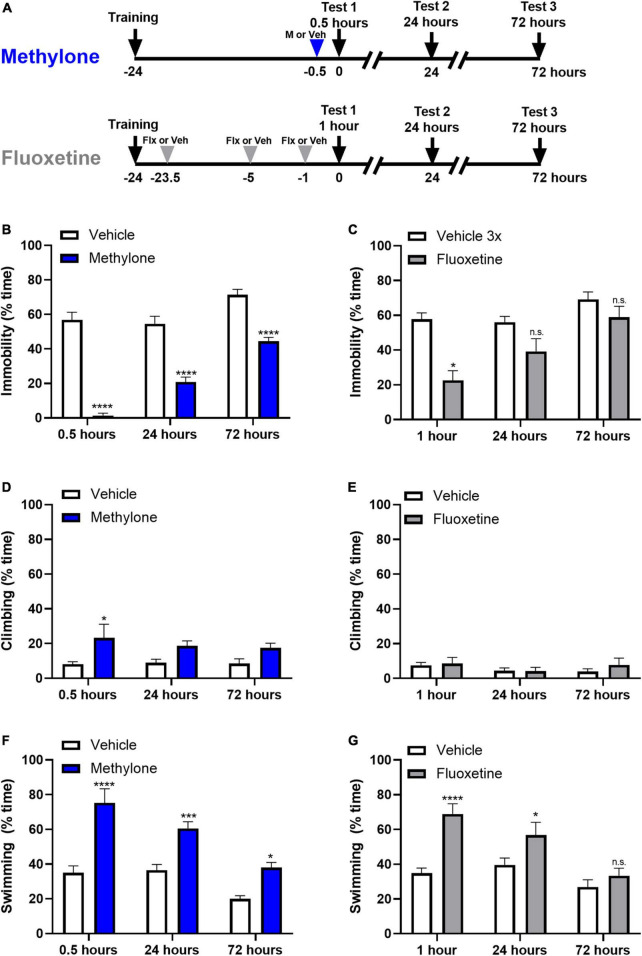
The effect of a single dose of methylone is long-lasting. **(A)** Schematic shows experimental design. Methylone (15 mg/kg, IP) or saline (Vehicle) was administered 30 min prior to forced swim testing. Fluoxetine (10 mg/kg, IP) or saline (Vehicle) were administered 23.5, 5, and 1 h prior to testing. Animals were retested 24 or 72 h later. Quantification of the percent time spent **(B,C)** immobile, **(D,E)** climbing, or **(F,G)** swimming during each 5-test session is shown. Data are presented as means ± SEM. *p < 0.05, ***p < 0.001, ****p < 0.0001 vs. Vehicle control group; n.s., not significant, Bonferroni’s *post-hoc* test; *N* = 6–8 per group.

In contrast to SAADs like fluoxetine, a feature of RAADs is that they produce more sustainable, longer-lasting effects that do not require daily dosing (22). To test whether this held true for methylone, animals were dosed once with methylone (15 mg/kg, IP) or saline (Vehicle) 30 min prior to testing in the FST and were retested 24 or 72 h later (Figure 3A). Methylone produced a durable antidepressant-like response, reducing immobility significantly at all-time points compared to vehicle controls [Figure 3B, *Drug*: *F*_(1,36)_ = 173.9 *p* < 0.0001; *Time*: *F*_(2,36)_ = 33.82, *p* < 0.0001; *Drug* × *Time*: *F*_(2,36)_ = 8.548, *p* < 0.001]. Fluoxetine (10 mg/kg, IP) or saline (Vehicle) were given 23.5, 5, and 1 h prior to testing, and rats were also retested 24 and 72 h later. In contrast to the durable effect of methylone, fluoxetine only showed a significant effect on immobility 1 h post-dose [Figure 3C, *Drug*: *F*_(1,36)_ = 25.85 *p* < 0.0001; *Time*: *F*_(2,36)_ = 11.94, *p* < 0.0001; *Drug* × *Time*: *F*_(2,36)_ = 3.328, *p* < 0.05]. A small but significant effect of methylone on climbing was observed at 30 min only [Figure 3D, *Drug*: *F*_(1,36)_ = 15.52 *p* < 0.001; *Time*: *F*_(2,36)_ = 0.3169, n.s.; *Drug* × *Time*: *F*_(2,36)_ = 0.4776, n.s.] and as expected, fluoxetine had no effect on climbing [Figure 3E, *Drug*: *F*_(1,36)_ = 15.52 *p* < 0.001; *Time*: *F*_(2,36)_ = 0.3169, n.s.; *Drug* × *Time*: *F*_(2,36)_ = 0.4776, n.s.]. Methylone had a more robust effect on swimming at this dose, consistent with the previous experiment, significantly increasing swimming at all three time points tested [Figure 3F, *Drug*: *F*_(1,36)_ = 64.93 *p* < 0.0001; *Time*: *F*_(2,36)_ = 21.12, *p* < 0.0001; *Drug* × *Time*: *F*_(2,36)_ = 3.786, *p* < 0.05]. Fluoxetine significantly increased swimming compared to vehicle controls, 1 h post-dose, and showed a small but significant increase in swimming 24 h post-dose [Figure 3G, *Drug*: *F*_(1,36)_ = 24.21 *p* < 0.0001; *Time*: *F*_(2,36)_ = 11.82, *p* = 0.0001; *Drug* × *Time*: *F*_(2,36)_ = 4.249, *p* < 0.05]. In summary, the antidepressant-like effect of a single dose of methylone lasted at least 72 h, whereas fluoxetine’s effect on immobility was only observed 1 h post-dose.

**FIGURE 4:**
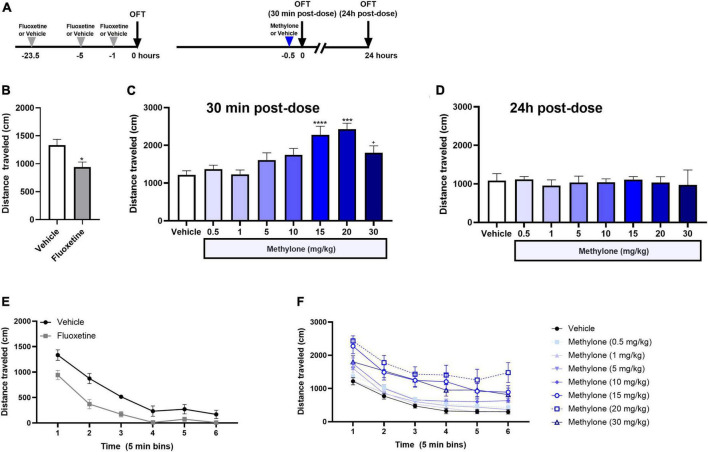
Effects of methylone on locomotor activity. **(A)** Schematic showing that the doses and time points tested in the OFT mirrored those used in FST dose response study. **(B)** Fluoxetine (10 mg/kg, IP) or saline (Vehicle) were administered 23.5, 5, and 1 h prior to testing, and the total distance traveled in the OFT is shown for the first 5-min of the test, corresponding to the testing duration in the FST. **(C)** Methylone (0.5–30 mg/kg, IP) or saline (Vehicle) was administered 30 min prior to testing, and the total distance traveled in the OFT is shown for the first 5 min of the test. **(D)** Effects of methylone on distance traveled for the first 5 min in the OFT were measured 24 h post-dose. Total distance traveled is shown for the duration of the 30-min OFT testing session in 5-min time bins after **(E)** fluoxetine or **(F)** methylone treatment. Data are presented as means ± SEM. *p < 0.05, ***p < 0.001, ****p < 0.0001 vs. Vehicle control group, Bonferroni’s *post-hoc* test *N* = 6 for fluoxetine; *N* = 7–13 per group for methylone. ^+^*p* = 0.08.

Since large changes in locomotor activity may confound the interpretation of results in the FST, we tested the effects of methylone or fluoxetine on locomotor activity in the OFT. First, we show effects on locomotion for the first 5 min in the OFT, corresponding to the 5-min FST testing session. Rats received fluoxetine (10 mg/kg) or saline (Vehicle) injections 23.5, 5, and 1 h before testing. A separate cohort of rats run in parallel received a single injection of methylone (0.5–30 mg/kg, IP) or saline (Vehicle) 30 min before testing in the OFT (Figure 4A). Fluoxetine, which reduces immobility in the FST, also reduced distance traveled in the OFT 1 h post-dose [Figure 4B, *t*_(10)_ = 2.882, *p* < 0.05], demonstrating that effects on activity do not always track with FST immobility. Higher doses of methylone (15–30 mg/kg) significantly increased distance traveled in the OFT, but lower doses of methylone (0.5–10 mg/kg), including two effective doses in the FST (Figure 1B), did not affect locomotor activity [Figure 4C, *F*_(7,72)_ = 5.816, *p* < 0.0001]. Rats were retested 24 h later, a time point at which the antidepressant-like effects of methylone persist (Figure 3B). Results showed that locomotor activity returned to baseline at this time point for all groups [Figure 4D, *F*_(7, 44)_ = 0.1450, n.s.]. Therefore, the antidepressant effects of methylone in the FST occur at doses (5–10 mg/kg) and time points (24 h) when there were no detectable changes in locomotor activity. Together, this supports that the antidepressant-like effect of methylone in the FST occurs independent of any changes in locomotion and that methylone has a transient stimulatory effect on distance traveled in the OFT.

The effect of methylone on the distance traveled in the OFT for the duration of a 30-min testing session is also shown, plotted in 5-min time bins, to determine whether methylone affected exploration or habituation to the testing chamber. Fluoxetine reduced distance traveled in the OFT for the first 15 min of the testing session [Figure 4E, *Drug: F*_(1,10)_ = 19.19, *p* < 0.01; *Time*: *F*_(3.286,32.86)_ = 87.29, *p* < 0.0001; *Time* × *Drug*: *F*_(5,50)_ = 2.353, *p* ≤ 0.05; *Subject: F*_(10,50)_ = 4.020, *p* < 0.001]. Higher doses of methylone (15–30 mg/kg) increased distance traveled for the duration of the testing session compared to saline-injected controls [Figure 4F, *Drug: F*_(7,72)_ = 6.080, *p* < 0.0001; *Time*: *F*_(2.995,217.7)_ = 200.6, *p* < 0.0001; *Time* × *Drug*: *F*_(35,360)_ = 1.566, *p* < 0.05; *Subject: F*_(72,360)_ = 22.50, *p* < 0.001].

### Effect of methylone on anxiety-like behavior

**FIGURE 5:**
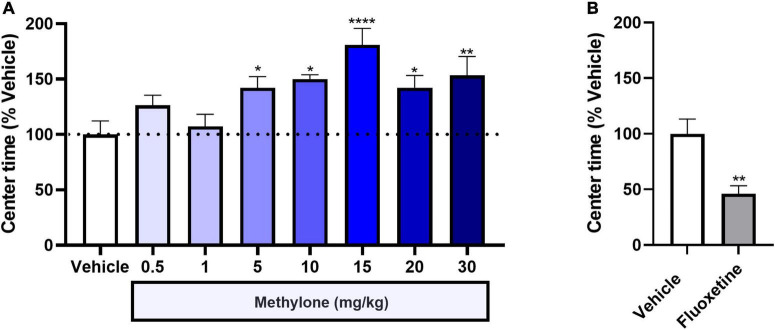
Methylone shows anxiolytic activity, increasing the time spent in the center of the open field test. The time spent in the center of the OFT is shown 30-min post-dosing with **(A)** methylone or **(B)** fluoxetine. Data are presented as means ± SEM. *p < 0.05, **p < 0.01, ****p < 0.0001, Fisher’s LSD *post-hoc* test; *N* = 6 for fluoxetine; *N* = 7–13 per group for methylone.

Here we investigated whether methylone also showed anxiolytic effects in a behavioral anxiety test, in addition to its antidepressant-like activity. Results revealed that methylone (5–30 mg/kg) significantly increased time spent in the center compared to controls 30 min after dosing [Figure 5A, *F*_(7,71)_ = 4.184, *p* < 0.001], consistent with an anxiolytic-like effect. Data are shown as the percent of vehicle control group, which on average, spent approximately 15 min in the center during the 30 min testing period. Fluoxetine significantly reduced time spent in the center [Figure 5B, *t*_(10)_ = 3.587, *p* < 0.01], consistent with an anxiogenic-like effect that has been described previously (25) and consistent with the anxiogenic effect of acute SSRI administration in humans. These results support the conclusion methylone has an acute anti-anxiety effect in addition to its antidepressant-like activity in the FST.

### Combined treatment effects of SSRI and methylone in the forced swim test

**FIGURE 6:**
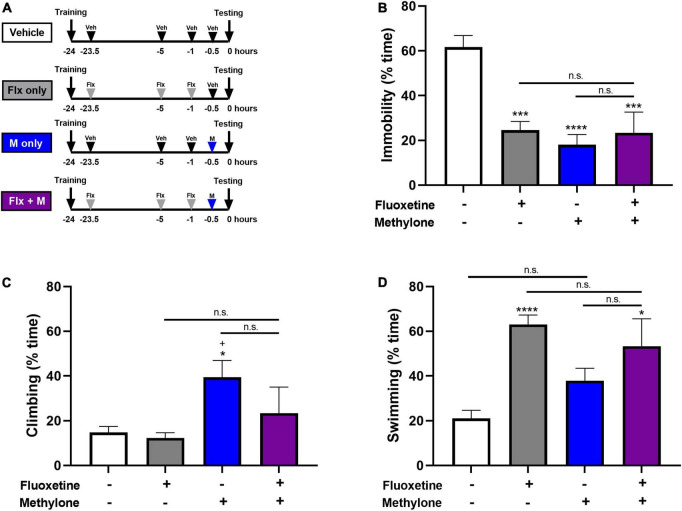
**(A)** Schematic shows experimental design and groups tested. Animals received three doses of fluoxetine (10 mg/kg, IP) 23.5, 5, and 1 h before testing and a single low dose of methylone (5 mg/kg, IP) 30 min before testing in the FST. Controls received fluoxetine only, methylone only, or saline vehicle. Quantification of the time spent **(B)** immobile, **(C)** climbing or, **(D)** swimming are shown. Data are presented as means ± SEM. *p < 0.05, ***p < 0.001, ****p < 0.0001 vs. Vehicle control group; ^+^*p* ≤ 0.05 vs. Fluoxetine alone, n.s., not significant, Bonferroni’s *post-hoc* test; *N* = 6–8 animals per group.

Since there is evidence of SSRIs interfering with the efficacy of MDMA in rodents and humans (7, 18, 26), we investigated whether combined treatment with fluoxetine interfered with the behavioral response to methylone in the FST. Fluoxetine (10 mg/kg, IP) or saline were administered 23.5, 5, and 1 h prior to testing, and a low but effective dose of methylone (5 mg/kg, IP) was administered 30 min prior to testing in the FST. Control groups received fluoxetine alone (10 mg/kg, IP), methylone alone (5 mg/kg, IP), or saline vehicle only (Figure 6A). A submaximal dose of methylone was chosen to allow for the possibility that fluoxetine might either augment or inhibit the methylone response. Results showed that fluoxetine had no effect on methylone’s activity in the FST. Compared to vehicle, all groups demonstrated a comparable reduction in immobility [Figure 6B, *F*_(3,26)_ = 13.53, *p* < 0.0001]. Methylone alone significantly increased climbing [Figure 6C, *F*_(3,26)_ = 3.947, *p* < 0.05] while fluoxetine and the combined treatment significantly increased swimming [Figure 6D, *F*_(3,26)_ = 8.677, *p* < 0.001]. In addition, we tested whether a maximally effective dose of methylone was affected by fluoxetine co-treatment, and found combined treatment with fluoxetine and a higher dose of methylone (15 mg/kg) also did not affect the response to methylone in the FST. Specifically, immobility was significantly reduced by 95% relative to the Vehicle control group (Methylone + Fluoxetine: 3.3 ± 2.4% time immobile vs. Vehicle: 61.6 ± 5.2, *p* < 0.0001), similar to the effect of methylone alone (4.2 ± 1.8% time immobile vs. Vehicle: 63.9 ± 2.7%, Figure 1B). In summary, the data demonstrate that combined treatment with fluoxetine had no effect on the response to methylone in the FST.

## Discussion

The current study shows for the first time that methylone has both antidepressant and anxiolytic effects in rodent tests of antidepressant-like activity and anxiety. Specifically, methylone had a rapid-acting, robust, and long-lasting antidepressant-like effect in the FST. The magnitude of the antidepressant effect observed after a single dose of methylone (i.e., 78–98% reduced immobility relative to Vehicle controls) was greater than many other antidepressants reported in the FST literature, including SSRIs sertraline (50–68%), paroxetine (40%), fluoxetine (56%), tricyclic antidepressant desipramine (55–75%), and RAADs ketamine (25–62%), MDMA (47–78%), and psilocybin (67%) (17, 19, 21–23). Moreover, and like other proposed RAADs such as ketamine, MDMA, and psilocybin, the effect of methylone was rapid-acting, occurring within 30 min of a single dose. In contrast, the effects of SSRIs and TCAs required three doses given between FST training and testing. Importantly, methylone’s effect in the FST is not due solely to stimulatory effects on locomotor activity. We further demonstrate an anxiolytic effect of methylone in the OFT. Pharmacokinetic profiling reveals dose-dependent changes in plasma and brain methylone concentrations and a brain-plasma ratio of approximately 1.8, all of which indicate CNS availability and are consistent with previous reports in rodents (27–29). Together, our results show that methylone may have potential to treat disorders such as MDD and anxiety. Future work in rodents and humans will aim to determine whether methylone may also have potential for treating PTSD and other CNS disorders for which conventional antidepressants are efficacious.

Despite having been synthesized over 25 years ago (9), there is a relatively small literature on methylone, and published studies have largely used binge-dosing regimens to mimic the illicit use of the drug. The only prior study of methylone in the FST reported that in mice, 3–4 doses of methylone (25 mg/kg) over a 2-day period increased immobility in the FST 3 days after dosing, consistent with a depression-like phenotype (29). This prior study intended to model the binge-dosing regimen of a drug user, as compared to our study, which aimed to mirror use of the drug at a lower, potentially therapeutic dose, accounting for the discrepancy in the findings. Similarly, while our data show that methylone is also anxiolytic in the OFT, two previous studies have shown anxiogenic effects of methylone using a binge-dosing regimen with dose levels and timing that differ significantly from the current study (30, 31).

There was a steep dose response relationship in immobility, such that the first significant effect was observed at 5 mg/kg and maximal effect at 10 mg/kg. We sought to determine whether pharmacokinetic properties of methylone could help to explain the observed behavioral responses. The plasma concentrations of methylone after 5 and 10 mg/kg doses were approximately 8 and 18 μM, respectively. The IC_50_ value for inhibition of serotonin uptake by methylone has been reported to be 5.75 μM *in vitro* (32). We speculate that at the 5 mg/kg dose, IC_50_ is likely achieved, and that at 10 mg/kg the transporter may become saturated, accounting for the maximal effect on behavior at this dose. However, additional studies, beyond the scope of the current report, are required to confirm this hypothesis.

Methylone is a structurally similar, beta-ketone analog of MDMA, so the similarities and differences between the two compounds should be considered when putting our new data into context. MDMA-assisted psychotherapy significantly alleviates symptoms of PTSD (7), a condition for which antidepressants (sertraline and paroxetine) are the only approved treatments (33). In contrast to methylone, MDMA has been well-studied using both binge-dosing paradigms and lower therapeutic doses in preclinical species. Both drugs produce antidepressant-like effects in the FST, specifically, MDMA has been shown to reduce immobility by 47–78% and methylone by 78–98% compared to vehicle controls (17, 23). Both are monoamine uptake inhibitors and releasers, but *in vitro* studies show that methylone is a 3–4-fold less potent inhibitor of serotonin uptake than MDMA (10, 28, 32, 34, 35). Serotonergic drugs like MDMA or SSRIs increase FST swimming behavior (19). Therefore, methylone’s lesser effect on serotonin can be observed in our FST data: lower doses of methylone increased climbing behavior, which has been linked to noradrenergic activity (19) and only at higher doses was swimming behavior, linked to serotonergic activity (19) increased. MDMA also disrupts vesicular stores of serotonin *via* vesicular monoamine transporter 2 (VMAT2), which contributes to its effects on serotonin release. In contrast, methylone has more than a 10-fold lower potency at VMAT2 than MDMA (32), suggesting methylone is more selective for monoamine transporters and that the psychopharmacology of these drugs occurs at the level of the monoamine transporters. Finally, repeated doses of methylone do not deplete brain serotonin like MDMA (10), suggesting that methylone may be more amenable to repeated clinical dosing at shorter intervals than MDMA, shortening the overall treatment duration.

Another consequence of MDMA’s effect on serotonin uptake and release is the potential interference of other drugs that have serotoninergic mechanisms, including SSRIs. Interference between the activities of MDMA and SSRIs when the drugs are co-administered has been reported previously in preclinical studies (36, 37). More recently, an analysis of phase 2 studies of MDMA-assisted psychotherapy showed that antidepressants that target monoamines, like SSRIs, reduce the efficacy of MDMA treatment (38), and exploratory analyses confirm a dampening of MDMA effectiveness by SSRIs (39), suggesting that patients should stop taking SSRIs before starting MDMA to gain the full benefit of the treatment. This is problematic because SSRIs are a first-line treatment for disorders like depression, anxiety, and PTSD, and it can take weeks or months to wean off of an SSRI, delaying treatment and risking serious worsening of patients’ symptoms in the interim. Co-treatment with an SSRI had no effect on methylone’s efficacy in the FST. Future studies in rodents and humans will determine whether SSRIs may be coadministered with methylone, as that would be a potential advantage of methylone over MDMA.

Methylone also had anxiolytic activity in a preclinical measure of anxiety, the OFT. A previous study showed there was no effect of MDMA on anxiety at clinically relevant doses, and that higher doses were anxiogenic (40), perhaps due to serotonin depletion (41). The anxiolytic activity of methylone observed here may be attributable to the fact that it does not deplete serotonin (10) and may be a serotonin receptor 1A (5-HTR1A) partial agonist (42), however, not all reports support activity at 5-HTR1A (43). Drugs with 5-HTR1A partial agonist activity, such as buspirone or aripiprazole, have anxiolytic effects and/or augment the efficacy of classical antidepressants, so this remains a direction of future study. Methylone is a monoamine uptake inhibitor and releaser. It acts as a substrate at plasma membrane transporters for dopamine (DAT), norepinephrine (NET), and serotonin (SERT) [reviewed by Baumann et al. (44)]. Overall, published reports suggest that methylone shows higher affinity for and greater effect on monoamine uptake inhibition at DAT and NET compared with SERT (32, 42, 43), distinguishing it from MDMA. In addition, methylone shows weak, if any, affinity for other 5-HT, NE, or DA receptors (43), suggesting that its primary mechanism may be through its actions at DAT, NET, and SERT. Our data provide the first behavioral evidence for the effectiveness of methylone in antidepressant and anxiety tests. Future work will address the underlying mechanism of action.

A limitation of the FST and its reliance on immobility as a primary outcome is that drugs that stimulate locomotion, like methamphetamine, can produce a false-positive result (45). It is notable, however, that there is often a dissociation between locomotor effects and immobility in the FST. For example, our data show that fluoxetine reduces immobility (increases swimming in the FST) but also reduces locomotor activity. Since methylone is a stimulant, reported previously to increase locomotor activity in rodents (46–49), it was important to investigate the effects of methylone on locomotor activity at all the doses used in the FST. Our results support the conclusion that the antidepressant effect of methylone is not driven solely by changes in locomotor activity because (1) lower doses of methylone (5–10 mg/kg) that are effective in the FST had no effect on locomotor activity in the OFT, and (2) 24 h after a higher dose (15 mg/kg), the antidepressant effect of methylone persisted while the locomotor effect resolved.

Antidepressant effects can differ depending on the emotional state of the animals being tested (50). Our studies were performed in naïve animals since the FST does not require repeated or chronic stress to elicit effects of antidepressants. Future studies will explore the effects of methylone in chronically stressed animals, and these experiments will help to extend our current findings and understand methylone’s effects in an animal model displaying deficits in emotional behavior.

The translatability of the FST has been questioned, despite its use for over 40 years to screen drugs with antidepressant-like activity (51, 52). However, we believe that it remains a useful screening tool. The translatability of our findings is supported by the reported positive clinical experience with methylone described in a recent retrospective case series of individuals with PTSD (13) and MDD (15). Using an allometric approach to dose scaling (53), rat doses are scaled to a human equivalent dose by dividing the rat dose (mg/kg) by 6.2 and multiplying by an average human bodyweight (estimated at 65 kg). Using this method, our data suggest that the most effective doses in the FST (10–15 mg/kg) correspond to human doses of approximately 100–150 mg. These doses have been evaluated in a phase 1 clinical study and are well tolerated, with no severe adverse events reported and with an effective but “gentler” subjective experience than MDMA (5, 11).

In conclusion, our results suggest that methylone may have a role in treating MDD and anxiety. Future studies in rodents and humans aim to determine whether methylone may also have potential for treating PTSD and other disorders for which antidepressants are effective.

## Data availability statement

The raw data supporting the conclusions of this article will be made available by the authors, without undue reservation.

## Ethics statement

This animal study was reviewed and approved by the Melior Discovery Institutional Animal Care and Use Committee (behavioral studies) and the WuXi AppTec Institutional Animal Care and Use Committee (pharmacokinetic studies).

## Author contributions

JW-S, MS, and SO conceived and designed the studies. JW-S analyzed the data and wrote the manuscript with comments from SO, MS, BM, CP, and BK. All authors contributed to the article and approved the submitted version.
